# Anti-inflammatory, antioxidant and antitumor activities of ingredients of Curcuma phaeocaulis Val

**DOI:** 10.17179/excli2015-231

**Published:** 2015-06-12

**Authors:** Yan Hou, Chuan-Li Lu, Qiao-Hui Zeng, Jian-Guo Jiang

**Affiliations:** 1 College of Food and Bioengineering, South China University of Technology, Guangzhou, 510640, China; 2 The Second Institute of Clinical Medicine, Guangzhou University of Chinese Medicine, Guangzhou 510120, China

**Keywords:** Curcuma phaeocaulis Val., anti-oxidant, anti-tumor, anti-inflammatory

## Abstract

*Curcuma phaeocaulis* Val. is used in Chinese Pharmacopoeia as health food and folk medicine for removing blood stasis, alleviating pain and tumor therapy. This research was aimed to explore and compare three main bioactivities including anti-oxidant, antitumor and anti-inflammatory activities between the ethanol extract of* C*.* Phaeocaulis* and its fractions using different *in vitro* models. Firstly, 70 % ethanol was used to extract* C. Phaeocaulis*, and then the crude extract was re-extracted, resulting in petroleum ether (EZ-PE), ethyl acetate (EZ-EA), and water fractions (EZ-W), respectively, and then a series of index was detected. Results showed that all the extracts had medium DPPH radical scavenging activity when the concentration was 200 μg/ml and their DPPH radical scavenging activity was in a concentration-dependent manner. The extracts except ethanol extract of *C. Phaeocaulis *had almost no cytotoxicity to the survival of RAW264.7 cell when the concentration reached 80 μg/ml, and all of them had medium inhibitory effect on nitrite release. Extracts of *C. Phaeocaulis *had medium intensity antitumor activity, EZ-PE and EZ-EA fractions significantly inhibited the proliferation of four tumor cells (SMMC-7721 cell lines, HepG-2 cell lines, A549 cell lines and Hela cell lines). *C. Phaeocaulis *had antioxidant and anti-inflammatory activities, which did not carry out centralized phenomenon when re-extracted. EZ-PE and EZ-EA were active antitumor sites of *C. Phaeocaulis*.

## Introduction

*Curcuma phaeocaulis* Val. belongs to the family Zingiberaceae. It is an important herbal drug prescribed in the Chinese Pharmacopoeia (Wang and Wang, 2001[21]). It is mainly distributed in the provinces of Guangxi and Sichuan in China (Sasikumar, 2005[16]). Its dried rhizomes have been used as health food and folk medicine with functions of removing blood stasis and alleviating pain (Wang and Wang, 2001[21]). In clinical practice, Rhizoma Curcumae is commonly prescribed for cardiovascular and tumor therapy alone or in combination with other herbs.

Surveys in India showed that Rhizoma Curcuma was one of the most commonly and popularly used medicinal plant for management of dermatological healthcare problems (Kumar et al., 2013[6]). The main bioactive constituents of Rhizoma Curcumae are essential oils, which possess anti-tumor (Wang et al., 2009[20]), anti-inflammatory (Makabe et al., 2006[8]), and neuroprotective properties (Dohare et al., 2008[2]). Different extracts have different constituents showing different biological activities. The water extracts of *C. phaeocaulis* showed relaxation effects while its polysaccharides induced contraction (Sasaki et al., 2003[15]). Water extract also displayed promoting learning, memory and anti-aging activity of mice (Mao et al., 2000[9]). The methanol extract of *C. phaeocaulis* was reported to have significant anti-inflammatory activity, which was manifested in its inhibitions on paw swelling, serum haptoglobin concentration, and cyclooxygenase-2 activity in adjuvant arthritis mice (Tohda et al., 2006[18]). The ethanol extract of *C. phaeocaulis* showed anti-tumor potential, which significantly inhibited MCF-7 cells proliferation by inducing apoptosis mediated by increasing ROS formation, decreasing Delta psi m, regulating BcI-2 family proteins expression, and activating caspases (Chen et al., 2011[1]). 

Previous studies mainly focus on a single active extract, the investigation of horizontal bioactivity comparison between the ethanol extract of *C. Phaeocaulis* and its fractions was little involved. Therefore, it is of great interest to test the antioxidant activity and other activities so that to develop novel promising and natural sources for antioxidants and functional foods. In the present research, we managed to figure out the antioxidant, anti-inflammatory, and anti-tumor activities of the ethanol extract of *C. Phaeocaulis* and its fractions (petroleum ether, ethyl acetate and water fractions), and then a comparative study between them was carried out.

## Materials and Methods

### Plant material

The dried rhizome of *C*.* phaeocaulis*, derived from Sichuan province, was purchased from Qingping medicinal material market (Guangzhou, China). A voucher specimen was deposited in the department of Natural Products Studies, School of Light Chemistry and Food Science, South China University of Technology. 

### Preparation of C. phaeocaulis extract 

The dried material was grounded in a cutting mill, then pass through an 100-mesh sieve to obtain a fine powder. All other reagents used in the experiment were of analytical grade. The powder (3.0 kg) of *C*.* phaeocaulis* was extracted with 95 % ethanol under reflux (3 × 7 L, each 2.5 h). The leach liquor was combined and concentrated under reduced pressure at 45 °C and the residue was reserved (EZ-Z, 77.8 g), which was suspended in water (1 L) and then partitioned with petroleum ether (3 × 1 L) and ethyl acetate (3 × 1 L) successively to give petroleum ether fraction (EZ-PE, 36.5 g), ethyl acetate fraction (EZ-EA, 28.7 g) and water remains (EZ-W, 9.4 g), respectively.

### DPPH radical scavenging assay

The DPPH radical scavenging effects of EZ-Z and its three fractions were detected according to the method of Roy et al. (2010[14]) with a bit modification. The DPPH solution was freshly prepared in methanol at a concentration of 1.75 × 10^−4^ mol/L. About 2.0 ml DPPH solution was added to 2.0 ml sample solution, and the mixture was vibrated for 20 s at room temperature. The absorbance of the mixture was recorded at 517 nm after reacting for 0.5 h in the dark. A control, in which the sample was replaced by methanol, was measured by the same way. DPPH radical-scavenging effect was calculated as follows:

*Radical - scavenging ratio (RSR, %) = (1-A_samp_ / A_contl_) x 100 %* [1] 

where A_contl_ is the absorbance value of the control group, and A_samp_ is the absorbance of the sample.

### Anti-inflammatory activity 

The anti-inflammatory activity assay was performed as described previously (Mitkus et al., 2013[12]). The mouse macrophage cell line RAW264.7 cells were cultured in Dulbecco's modified Eagle medium (DMEM) supplemented with 10 % heat-inactivated fetal bovine serum (FBS), 1 % penicillin-streptomycin and maintained in an atmosphere of 5 % CO_2_ at 37 °C. RAW264.7 cells (5 × 10^5^ cells/ml) were seeded in 96-well culture plates (100 μl/well) and then incubated with or without lipopolysaccharide (LPS, final concentration 1 μg/ml) in absence or presence of samples with various concentrations (6.25, 12.5, 25.0, 50.0, 100.0 μg/ml) for 24 h. The nitrite accumulated in culture medium was measured as an indication based on the Griess reaction. 100 μl of culture medium was mixed with 100 μl Griess reagent [equal volumes of 1 % (w/v) sulphanilamide in 2.5 % (v/v) phosphoric acid and 0.1 % (w/v) N-1-naphthylenediamine dihydrochloride]. The absorbance of mixture at 540 nm was measured 10 min later and calibrated using a standard curve of sodium nitrate prepared in culture media (Mathew and Sharma 2000[10]; Meli et al., 2000[11]).

### Evaluation of anti-tumor effects

The anti-tumor activity of EZ-Z and its three fractions (EZ-PE, EZ-EA and EZ-W) were tested by MTT [3-(4,5-dimethylthiazol-2yl)-2,5-diphenyltertrazoliumbromide] assay with HepG-2, A549, SMMC-7721 and Hela cell lines (Smith et al., 1998[17]; Liu et al., 2012[7]). Cells were all obtained from Sun Yat-Sen University, Guangzhou, China, and were cultured in DMEM medium supplemented with 10 % heat-inactivated FBS, penicillin (100 U/ml) and streptomycin (100 μg/ml) under an atmosphere of 5 % CO_2_ at 37 °C. 100 μl exponentially growing cells (5 × 10^4^ cells/ml) were seeded in 96-well plates and cultured for 12 h. Then 100 μl sample solution with different concentrations were added to each well for 24 h at 37 °C. Positive controls were treated with the same amount of 5-Fluorouracil (5-F). Blank controls were treated with DMEM medium without any sample. Optical density (OD) at 570 nm was used as a measure of cell viability. Cell survival rate (%) was calculated by the following formula:

*Cell survival (%) = (OD_contl_ - OD_samp_) / OD_blank_ x 100 %* [2] 

where OD_contl_, OD_samp_ and OD_blank_ were the optical density at 570 nm of the 5-F, sample and blank group, respectively.

### Statistic analysis 

Each experiment was performed in triplicate, and the data were expressed as mean ± SD. The significance of differences between groups was assessed by one-way analyses of variance (ANOVA). P < 0.05 indicated the presence of a statistically significant difference and P < 0.01 was considered highly significant. 

## Results and Discussion

### Antioxidant activity 

Radical-scavenging activity (RSA) assay has been widely used to evaluate the antioxidant effects of natural drugs (Ghazanfari et al., 2006[3]; Hwang et al., 2013[5]; Vulic et al., 2013[19]; Hatamnia et al., 2014[4]), Seeing that the laboratory-generated free radical such as hydroxyl radical and superoxide anion could be easily affected by some side reactions, such as metal-ion chelation and enzyme inhibition brought about by various additives, while DPPH has no these shortages, here we use DPPH radical scavenging assay to evaluate the antioxidant effects of the ethanol extract of* C*.* Phaeocaulis* and its fractions (petroleum ether, ethyl acetate and water fractions). 

Newly prepared DPPPH solution exhibits a deep purple color and it has maximum absorption at 517 nm, when antioxidant was added, the color generally fades or disappears, and the absorption would change. The reason of the change in color and absorption was mainly because antioxidant molecules can quench DPPH free radicals (i.e. by providing hydrogen atoms or by electron donation, conceivably via a free-radical attack on the DPPH molecule) and convert them to a colorless/bleached product (i.e. 2, 2-diphenyl-1-hydrazine, or a substituted analogous hydrazine) (Yamaguchi et al., 1998[22]). Therefore, when a substance make the absorbance of DPPH solution decrease, it can be thought to possess the antioxidant activity, and the faster the absorbance decreases, the stronger antioxidant activity the extract have.

**Figure 1:**
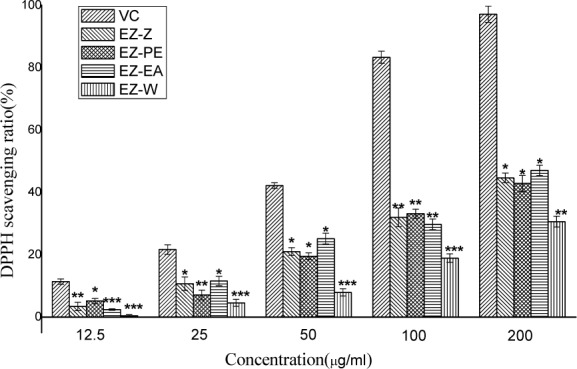
DPPH radical scavenging activity of the ethanol extract of *C*.* phaeocaulis* and its sub-fractions. Results are mean ± SD. *P < 0.05, **P < 0.01, statistically significant in comparison with the control (V_C_)

The DPPH scavenging effect of the crude ethanol extract and its four fractions were tested and compared with each other. Figure 1(Fig. 1) reflects the dose-response relationship of extracts; the results were expressed as a percentage of the ratio of the decrease in absorbance at 517 nm to the absorbance of DPPH solution without samples at 517 nm (Yoshida et al., 1989[24]). When the concentration was 200 μg/ml, the EZ-EA afforded greatest RSA on the stable DPPH free radical, measuring 47.04 %, followed by EZ-Z, EZ-PE, EZ-W at 44.66, 42.84 and 30.58 %, respectively. 

All extracts exhibited DPPH radical scavenging activity in a concentration-dependent manner that radical scavenging ratio was rising with the increase of sample concentration. Petroleum ether fraction and ethyl acetate exhibited similar radical scavenging ability with ethanol extract of* C*.* Phaeocaulis*, indicating that antioxidant activity did not carry out centralized phenomenon when ethanol extract of* C*.* Phaeocaulis* had been re-extracted.

### Anti-inflammatory activity

**Figure 2:**
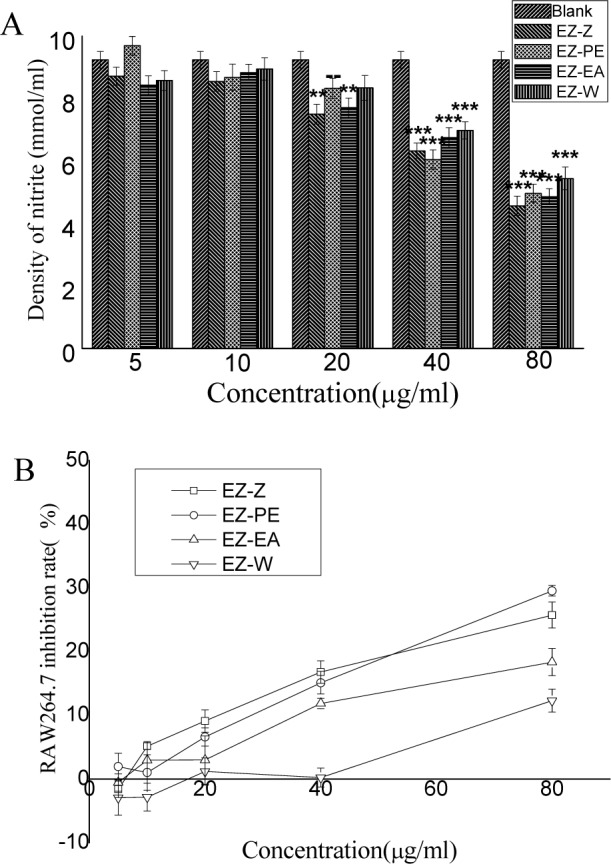
(A) Inhibitory effect on nitrite production of the ethanol extract of *C. phaeocaulis *and its sub-fractions in LPS-induced RAW264.7 macrophages; (B) Cytotoxic effect of the ethanol extracts and their polar fractions of *C. phaeocaulis* on RAW264.7 cell lines. Results are mean ± SD. *P < 0.05, **P < 0.01, ***P < 0.001, statistically significant in comparison with the others

Current studies have demonstrated the participation of reactive oxygen species in models of inflammation. *C*.* Phaeocaulis* was investigated as potential inhibitors of nitrite production in inflammatory reactions. Stimulation of RAW264.7 macrophages by LPS-induced lead to overproduction of nitrite, which could be detected and quantified. Results presented in Figure 2A(Fig. 2) showed that all extract and fractions significantly inhibited nitrite release and the release of nitrite decreased in the order of EZ-W (5.44 mmol/ ml), EZ-PE (4.96 mmol/ml), EZ-EA (4.86 mmol/ml) and EZ-Z (4.57 mmol/ml) at the concentration of 80 μg/ml. It was noted that EZ-Z exhibited the most active anti-inflammatory effect among all extract and fractions. Many previous literatures showed that methanol/ethanol extracts or fractions had good anti-inflammatory activity. Yang et al. (2013[23]) reported that ethyl acetate fraction of the seeds of *Brucea Javanica* showed significant decrease on nitrite production in LPS-induced RAW264.7 macrophages. In this study, EZ-Z and EZ-EA both showed a better anti-inflammatory ability than other fractions. 

Results presented in Figure 2B(Fig. 2) showed that the ethanol extract of *C. phaeocaulis *exhibited a certain cytotoxicity, and the survival rate of RAW 264.7 cells decreased from 94.85 to 74.3 % with the concentration increased from 10 to 80 μg/ml. EZ-PE and EZ-EA exhibited similar intensity cytotoxicity with EZ-Z. EZ-W did not exhibited obvious cytotoxicity, as its cells survival was greater than 87 % even at the highest concentration (80 μg/ml). 

Altogether, these results suggest that the ethanol extract of *C. phaeocaulis *had some anti-inflammatory activity at certain concentration. Since active chemical did not carry out centralized phenomenon when ethanol extract of* C*.* Phaeocaulis* had been re-extracted, its three sub-fractions exhibited only a certain anti-inflammatory.

### Anti-tumor

**Figure 3:**
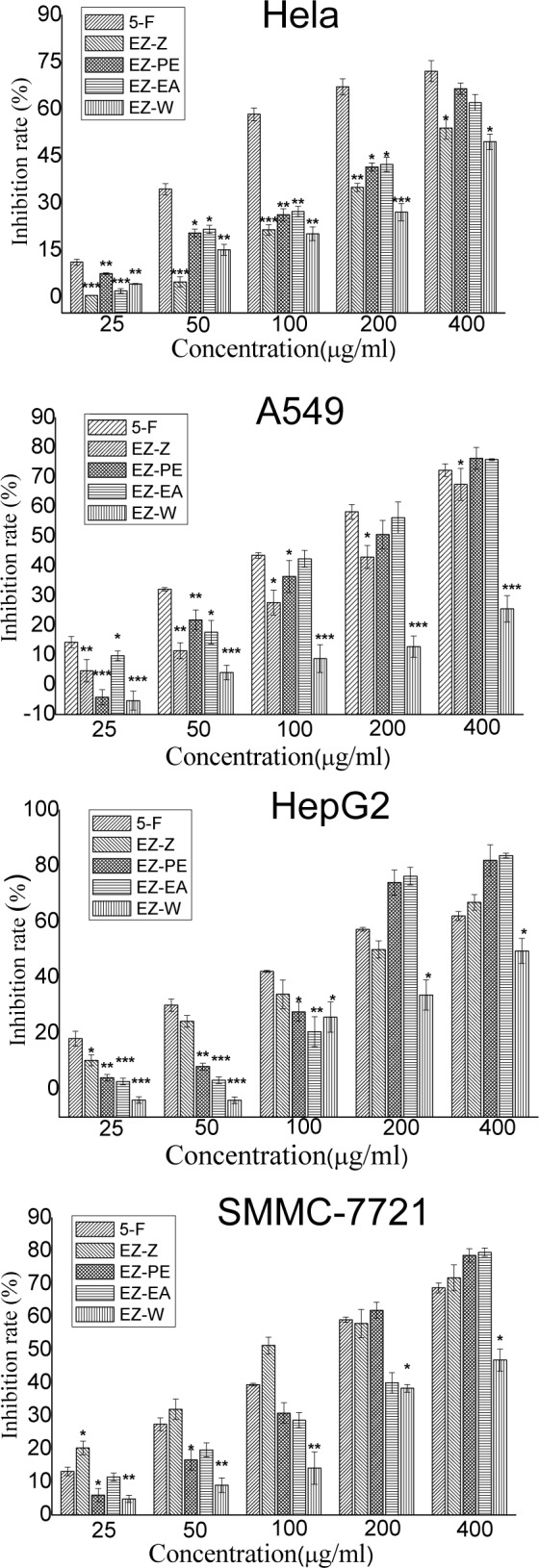
Inhibition of the ethanol extracts and its polar fractions of C. phaeocaulis on Hela, HepG-2, SMMC-7721 and A549 cells. Results are mean ± SD. *P < 0.05, **P < 0.01, ***P < 0.001, statistically significant in comparison with the control (5-fluorouracil)

The antitumor activity of above samples was also determined by MTT assay. Figure 3(Fig. 3) shows the cell proliferation inhibition rate of each sample. We can see from the figure that the ethanol extract of *C. Phaeocaulis *exhibited medium intensity proliferation inhibition effect on four tumor cells, and the effect was in a concentration- depend manner. For Hela cell lines, EZ-PE and EZ-EA both exhibited medium cytotoxicity, and their inhibition rate were higher than EZ-Z and EZ-W. For HepG-2 cell lines, the proliferation inhibition effect of EZ-PE and EZ-EA was significantly higher than EZ-Z and EZ-W at the concentration ≥ 200 μg/ml, while this effect was lower than EZ-Z and EZ-W at the concentration < 200 μg/ml. For SMMC-7721 cell lines, the antitumor activity of three polar extracts of *C. phaeocaulis *was significantly lower than its ethanol extract, indicating that the active components were scattered after extraction. For A549 cell lines, when the concentration ≥ 50 μg/ml, the antitumor activity order of four fractions was ethyl acetate fraction ≥ petroleum ether fraction > ethanol extract > water fraction. 

**Figure 4:**
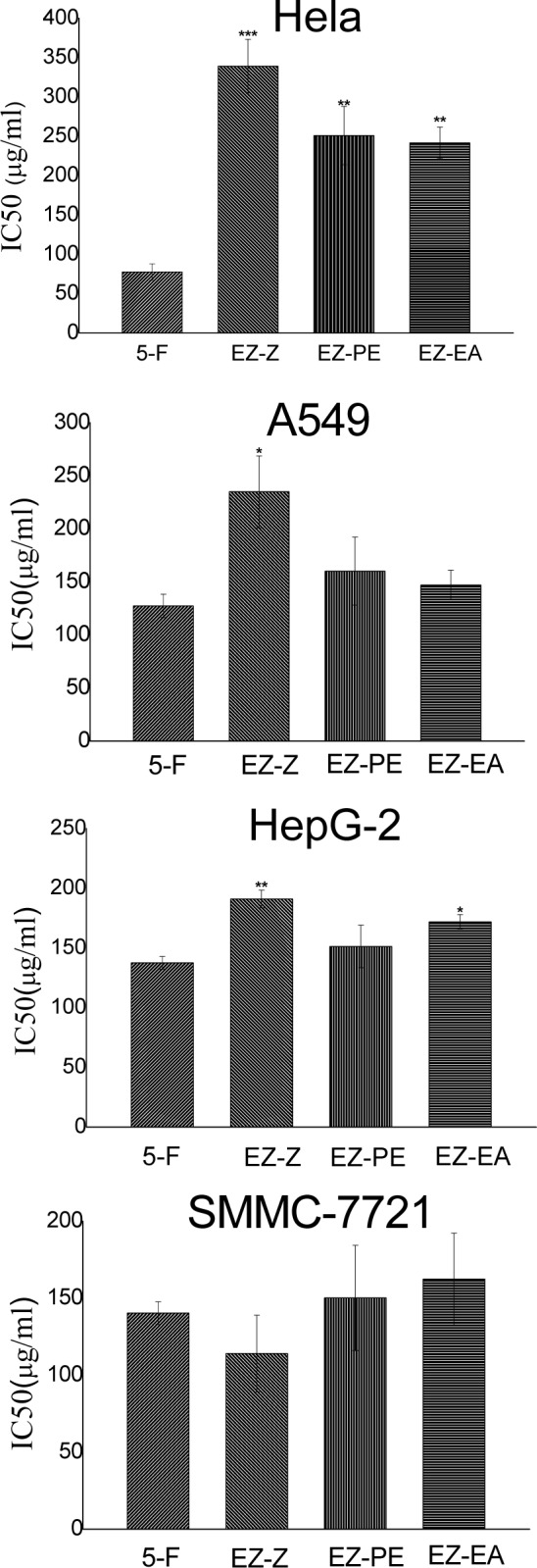
Comparison of half maximal (50 %) inhibitory concentration (IC_50_) between various fractions of *C. phaeocaulis *on Hela, HepG-2, SMMC-7721 and A549 cells. Results are mean ± SD. *P < 0.05, **P < 0.01, ***P < 0.001, statistically significant in comparison with the control (5-fluorouracil)

IC_50_ value usually used as a measure of drug effectiveness, which was calculated by regression (curve fitting) of a series cell viability data, it is a value of the drug concentration at which 50 % of the cell population in a designated period was destroyed (Muthu et al., 2011[13]). The IC_50_ of all fractions were shown in Figure 4(Fig. 4). Considering that the water fraction had little effect of antitumor, the IC_50_ value of water fraction was not calculated. For Hela cell lines, the IC_50_ of EZ-EA fraction was the lowest, 255.2688 μg/ml, For HepG-2 cell lines, the IC_50_ of EZ-PE fraction was the lowest, 132.6822 μg/ml, For SMMC-7721 cell lines, the IC_50 _of EZ-EA fraction was the lowest, For A549 cell lines, the IC50 of EZ-Z fraction was the lowest, 111.0659 μg/ml.

These data suggests that extracts of *C. phaeocaulis *had medium intensity antitumor activity and petroleum ether fraction and ethyl acetate fraction had antitumor activity for some tumor cell lines after re-extracted, indicating that petroleum ether fraction and ethyl acetate fraction were active site of *C. phaeocaulis*, which is in accordance with Radical-scavenging activity result. All these facts illustrated that radical scavengers may protect cell tissues from free radicals, thereby preventing diseases such as cancer (Young-Joon, 2002[25]). 

In conclusion, petroleum ether and ethyl acetate fraction exhibited similar radical scavenging ability with ethanol extract of* C*.* phaeocaulis*, as determined by scavenging effect on the DPPH· free radical. The ethanol extract of *C. phaeocaulis *had some anti-inflammatory activity at certain concentration, and its three sub-fractions also exhibited similar anti-inflammatory, indicating that antioxidant and anti-inflammatory active chemicals were distributed in four parts when re-extracted. The ethanol extracts of *C. phaeocaulis *had medium intensity antitumor activity and petroleum ether fraction and ethyl acetate fraction had stronger antitumor activity for some tumor cell lines after extracted, indicating that petroleum ether fraction and ethyl acetate fraction were active site of *C. phaeocaulis*. These data can provide some scientific basis for the further study on the antitumor activity of* C. phaeocaulis*. In future, animal experiments *in vivo* should be performed to further conform the antitumor activity of the fractions and to elucidate their related underlying mechanism.

## Notes

Yan Hou and Chuan-Li Lu contributed to the work equally and should be regarded as co-first authors.
